# On-The-Go VIS + SW − NIR Spectroscopy as a Reliable Monitoring Tool for Grape Composition within the Vineyard

**DOI:** 10.3390/molecules24152795

**Published:** 2019-07-31

**Authors:** Juan Fernández-Novales, Javier Tardáguila, Salvador Gutiérrez, María Paz Diago

**Affiliations:** 1 University of La Rioja, Department of Agriculture and Food Science, 26006 Logroño, Spain; 2 Institute of Grapevine and Wine Sciences (University of La Rioja, Consejo Superior de Investigaciones Científicas, Gobierno de La Rioja), 26007 Logroño, Spain; 3 Department of Computer Science and Engineering, University of Cádiz, Avda, de la Universidad de Cádiz 10, 11519 Puerto Real, Cádiz, Spain

**Keywords:** *Vitis vinifera* L., proximal sensing, precision viticulture, near infrared, chemometrics, non-destructive sensor

## Abstract

Visible-Short Wave Near Infrared (VIS + SW − NIR) spectroscopy is a real alternative to break down the next barrier in precision viticulture allowing a reliable monitoring of grape composition within the vineyard to facilitate the decision-making process dealing with grape quality sorting and harvest scheduling, for example. On-the-go spectral measurements of grape clusters were acquired in the field using a VIS + SW − NIR spectrometer, operating in the 570–990 nm spectral range, from a motorized platform moving at 5 km/h. Spectral measurements were acquired along four dates during grape ripening in 2017 on the east side of the canopy, which had been partially defoliated at cluster closure. Over the whole measuring season, a total of 144 experimental blocks were monitored, sampled and their fruit analyzed for total soluble solids (TSS), anthocyanin and total polyphenols concentrations using standard, wet chemistry reference methods. Partial Least Squares (PLS) regression was used as the algorithm for training the grape composition parameters’ prediction models. The best cross-validation and external validation (prediction) models yielded determination coefficients of cross-validation (R^2^_cv_) and prediction (R^2^_P_) of 0.92 and 0.95 for TSS, R^2^_cv_ = 0.75, and R^2^_p_ = 0.79 for anthocyanins, and R^2^_cv_ = 0.42 and R^2^_p_ = 0.43 for total polyphenols. The vineyard variability maps generated for the different dates using this technology illustrate the capability to monitor the spatiotemporal dynamics and distribution of total soluble solids, anthocyanins and total polyphenols along grape ripening in a commercial vineyard.

## 1. Introduction

Grape berry ripening is usually described as the accumulation of sugars, and it is measured in terms of total soluble solids (°Brix). However, there are other compositional variables taken into account to determine the optimal maturity for harvest, such as berry acidity, often expressed as pH, titratable acidity and concentrations of tartaric and malic acids, berry weight, as well as the anthocyanin and total phenol concentrations (in red varieties) [1]. In red grape varieties, anthocyanins and other polyphenols are located in berry skins, and their accumulation starts at veraison and continues during ripening [2]. Anthocyanins are red coloured phenolic compounds or pigments, which are responsible for the red wine colour [3], while the other phenolic compounds, such as the flavonols, flavanols, hydroxycinammic acids, and stilbenes may increase and stabilize the wine color by means of the copigmentation phenomenon [4,5] and contribute to the wine’s mouthfeel and taste perception attributes [6]. Both the anthocyanin and total phenolic contents in berries are currently measured using ‘wet chemistry’ procedures [7,8], which are time-consuming and labour-intensive, while the total soluble solids (TSS) are more easily measured using a refractometer. Both methodologies are destructive and require time-consuming berry sampling and sample preparation (for anthocyanins and total polyphenols content), which hinder their massive application (analysis of a large number of samples) to assess the spatial variability of grape composition within a given vineyard plot at a specific date. The occurrence of spatial variability of grape compositional parameters in many vineyards is nothing new, and most widely used berry sampling procedures [9,10] do attempt to have it into consideration when defining the trajectories and manual sampling protocols within a plot. However, these same procedures end up with a berry sample of 100 to 200 berries or 20 to 40 clusters, regardless the size of the plot, in most cases. Should we consider a 1.0 ha vineyard plot, the estimated number of total berries could be well beyond 4–5 million berries. Therefore, a 200-berry sample, even if it is picked across many rows, barely represents less than ~0.005% of the berry production in this 1.0 ha plot, whose analytical measurements are often used to drive decisions on harvest scheduling or grape quality classification and pricing, in some cases.

Within the context of precision agriculture, the development of new sensors, especially based on spectroscopy, enables high resolution data acquisition that could be used to track crop development and ripening. In this regard, the capability to assess ripening in a fast, non-destructive way would substantially and positively impact the process of harvest scheduling and classification.

Visible and near-infrared (Vis-NIR) spectroscopy is a well-known technique for the non-destructive measurement of quality attributes of fruits and vegetables [11,12]. The Vis-NIR region covers the range of the electromagnetic spectrum between 380 and 2500 nm. Spectroscopy techniques combined with multivariate analysis have been widely used for quantitative determination of several quality properties or chemical compounds in fruit, to determine ripeness, and to measure quality indices [13,14,15,16,17,18,19,20].

In the context of grape composition monitoring during the ripening process using NIR spectroscopy, a variety of works has been published [21,22,23,24,25], but all these were conducted under controlled conditions, such as illumination, temperature, humidity, and sample positioning, among others-i.e. in a laboratory. In-field measurements have deserved less attention, and fewer number of works have addressed the utilization of manual, portable, hand-held spectrometers to assess the composition of grape berries while they are still on the grapevines [26,27,28].

One step forward has been recently given by Gutiérrez et al. (2019) [29] who reported the quantification of TSS and anthocyanins in grape berries under field conditions, using on-the-go hyperspectral imaging (HSI) between 400 and 900 nm, acquired from a moving platform. HSI is a very powerful technology, capable of recording the whole spectrum within a given spectral range in each specific pixel of a two-dimensional image. Its potential to yield a huge amount of relevant information is well recognized, but the difficulties in analyzing this information are also HSI’s main drawback.

On-the-go VIS-NIR spectroscopy has been successfully used to assess the grapevine water status [30,31]. Using this technology, an average spectrum of a circular measuring spot with a diameter of ~1.9 cm is acquired at a rate of acquisition between 15 to 28 measurements per second, which provides a sufficient amount of spectral measurements, whose analysis is less complex than that of HSI.

Therefore, given the good prediction results that manual VIS-NIR spectroscopy demonstrated under control conditions, and the experiences of on-the-go VIS-NIR spectroscopy and HSI in the vineyard, the aim of the present work was to assess and map the grape composition parameters (TSS, anthocyanins content and total polyphenols) along the ripening period using a proximal VIS + SW-NIR sensor from a motorized platform in the vineyard.

## 2. Results

### 2.1. Berry Composition

**Figure 1:**
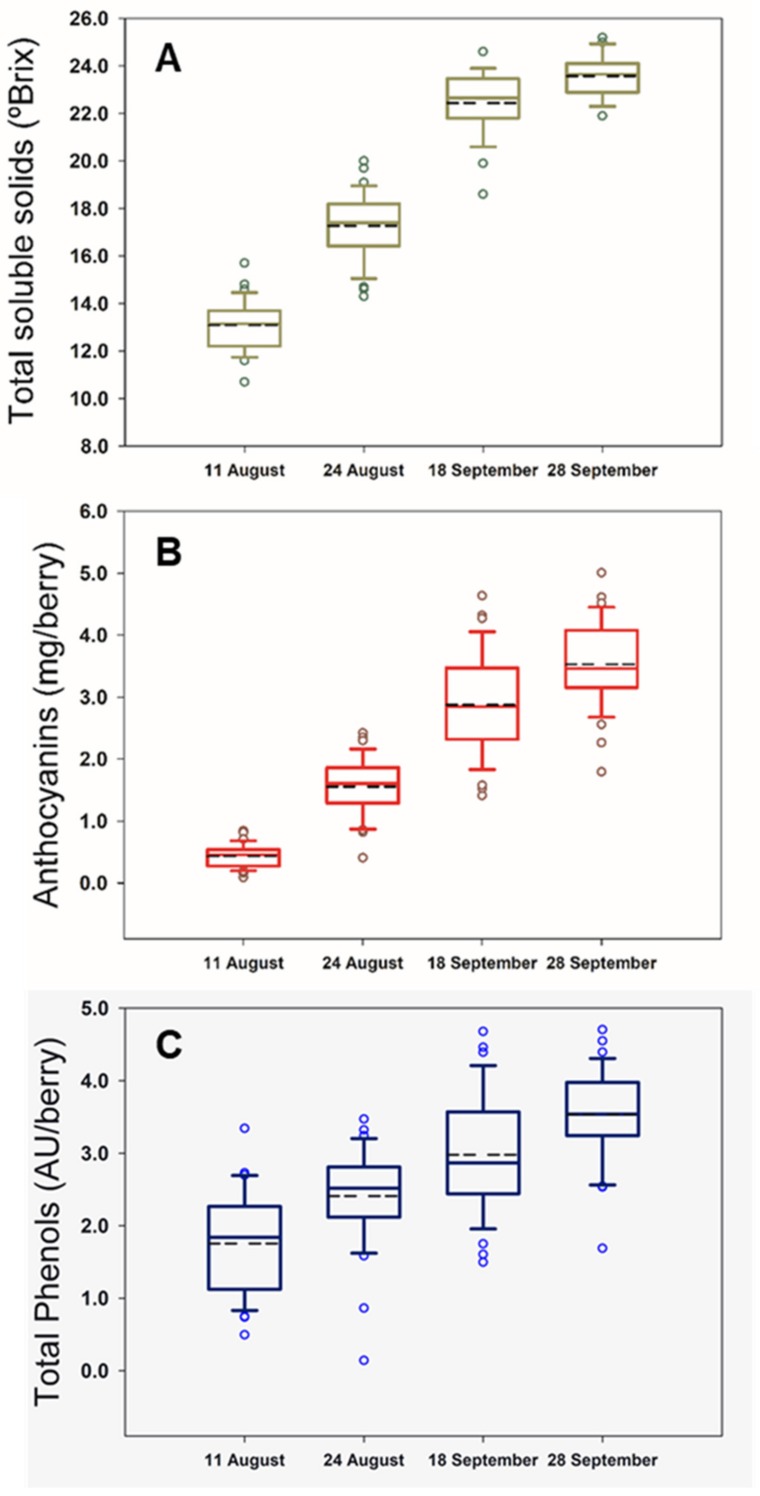
Box plots for total soluble solids (**A**), anthocyanins (**B**), and total polyphenols (**C**) during the four dates of the experimental study. Dashed lines represent mean values.

The boxplots for the berry composition analysis in four different dates throughout the field experiment are shown in Figure 1. This type of graphs provides a convenient way of visually displaying the data distribution through their quartiles. The boxplots in Figure 1 are not skewed indicating that the data were normally distributed. Moreover, they illustrate the different ripening rates among grapevines and inherent variability per measurement date within the vineyard for the monitored compositional variables, such as TSS (Figure 1A), total anthocyanins (Figure 1B) and polyphenols (Figure 1C).

The studied parameters were well represented with an adequate variability. Likewise, TSS varied between 10.7 °Brix to 25.2 °Brix, while anthocyanins and total polyphenols ranged from 0.09 mg/berry and 0.14 AU/berry to 4.64 mg/berry and 4.70 AU/berry, respectively. The mean values increased throughout the season as illustrated in Figure 1.

### 2.2. Regression Models and Mapping for Grape Composition Parameters

The performance statistics of the best regression models of calibration, cross-validation, and external validation (prediction) for the prediction of TSS, anthocyanins and total polyphenols in grape clusters under field conditions from on-the-go Vis + SW − NIR spectroscopy are summarized in Table 1.

Diverse pre-processing operations were applied. However, the best models involved the implementation of the Savitzky–Golay first derivative and a window size of 15. In the case of total polyphenols, the standard normal variate (SNV) filtering was also applied for spectra pre-processing. No anomalous spectra were identified following the Residuals (Q) and Hotelling values (T^2^) and, moreover, the models were developed with low number of latent variables (Table 1), which calls for increased robustness and higher capability of generalization.

The best models for cross and external validations (also called prediction) returned determination coefficient values higher than 0.90 for TSS (R^2^_cv_ = 0.92, R^2^_p_ = 0.95), higher than 0.75 for anthocyanins (R^2^_cv_ = 0.75, R^2^_p_ = 0.79) and more modest values for total polyphenols (R^2^_cv_ = 0.42, R^2^_p_ = 0.43). The accuracy of the models in terms of RMSECV and RMSEP values were below 1.24 °Brix for TSS, 0.664 mg/berry for anthocyanins and 0.749 AU/berry for total polyphenols, respectively (Table 1).

**Figure 2:**
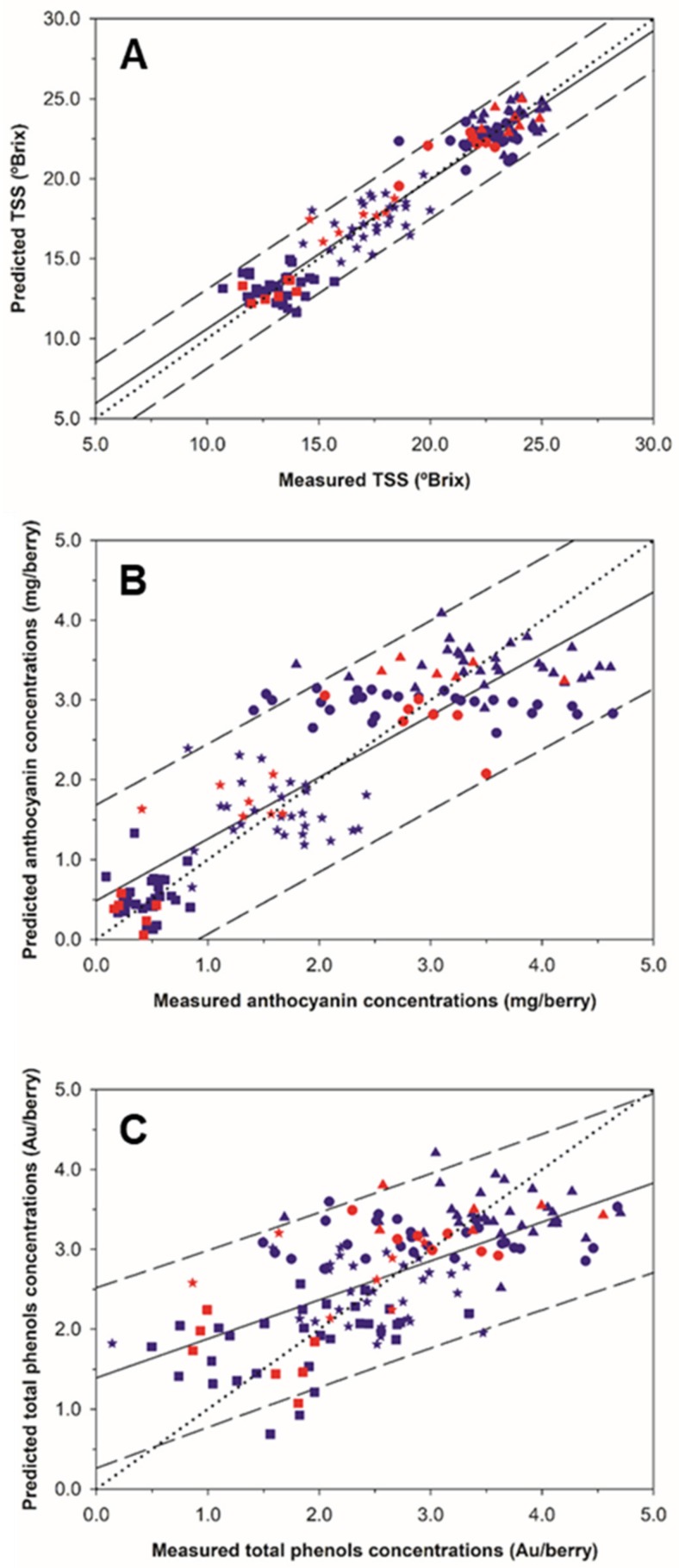
Regression plots for the total soluble solids (**A**), anthocyanins (**B**) and total polyphenols (**C**) using the best Partial Least Squares (PLS) models generated from on-the-go grape clusters spectral measurements. (blue color) 10-fold cross validation; (red color) external validation. (■: 11 August; *: 24 August; ●: 18 September; ▲: 28 September). Solid line represents the regression line and dotted line refers to the 1:1 line. Prediction confidence bands are shown at a 95% level (dashed lines).

Figure 2 shows the regression plots for the best prediction models for TSS, anthocyanins and total polyphenols in grape clusters under field conditions from on-the-go spectral measurements. A greater scattering of the points around the regression line was observed for the TSS in comparison with the other two parameters. All the samples from the TSS regression model exhibited a very good fit along the correlation line and were also inside of the 95% confidence bands, except three of them (Figure 2A).

A wide data range was covered by the samples from anthocyanins and total polyphenols regression models. The 1:1 line displayed a better fit over the anthocyanins regression line (Figure 2B) than over the total polyphenols regression line (Figure 2C) Additionally, seven out of 144 samples lied outside the anthocyanins prediction bands, keeping 95.14% of the samples (Figure 2B), while for total polyphenols 93.75% of the samples lied within prediction bands (Figure 2C).

**Figure 3:**
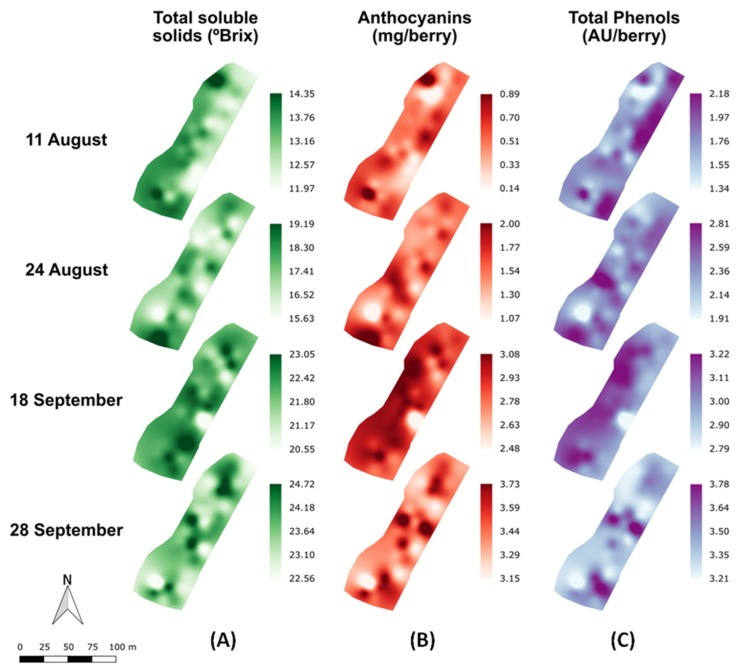
Prediction maps of the spatial variability of anthocyanins (**A**), total soluble solids (**B**), and total polyphenols concentrations (**C**) along the grape ripening period (11 August to 28 September).

To analyze the spatial variability of the grape composition parameters in a commercial vineyard along the different maturity stages (11 August to 28 September), maps for TSS, anthocyanins and total polyphenols were computed and presented in Figure 3. The highest values of TSS for each stage were mainly found at the north and south areas of the vineyard plot, while in the central part of the plot generally lower values were detected.

The general evolution trend along the vineyard of anthocyanins and total polyphenols concentrations showed a different pattern than that of TSS, but very similar between them. Along the four measuring dates, a monotonous increase of the anthocyanin and total phenol concentration was observed. Since the anthocyanins largely contribute to the phenolic pool of compounds in the berries the similarity between their spatiotemporal evolution from veraison to harvest is coherent.

## 3. Discussion

The results presented in this work have demonstrated the capability of contactless VIS + SW − NIR reflectance spectroscopy in the range of 570–990 nm acquired on-the-go, from a motorized platform in the field, to estimate key parameters of grape composition. To achieve this, robust and reliable prediction models were generated for three important grape composition indicators from spectra of grape clusters acquired non-destructively from a moving vehicle at a speed (5 km/h) similar to that used for conventional machine operations. Furthermore, on-the-go VIS-SW-NIR spectroscopy has also been successful in characterizing the spatiotemporal dynamics of the accumulation of total soluble solids, anthocyanins and total polyphenols along ripening, within a commercial vineyard.

Many authors have reported that the monitoring of grape quality non-destructively through the ripening process under laboratory conditions is predominantly represented by two vibrational spectroscopy-related technologies: Near infrared spectroscopy [21,22,23,24,25] and hyperspectral imaging [32,33,34,35]. Nevertheless, little research has been conducted directly in the field using NIR spectroscopy [26,27,28]. In these works, a contact portable instrument was used to determine TSS in different red varieties, reporting prediction RMSE values of 1.25, 1.24, and 1.68 °Brix, respectively. In terms of the anthocyanin concentrations, [27] reported values of R^2^_v_ = 0.624, RMSEV = 0.302 mg/g using the spectral range between 640 and 1300 nm. These performance numbers are in good agreement with the RMSEP values obtained in the present work, although in this case, spectral acquisition was carried out contactless, on-the-go, from a moving vehicle along the vineyard.

Very recently, the capability of in-field, on-the-go hyperspectral imaging for the assessment of total soluble solids and anthocyanin concentrations in wine grapes in a commercial vineyard has been tested [29]. In this study, the performance of the models obtained with support vector machines for TSS and anthocyanin concentrations returned determination coefficients for external prediction R^2^ of 0.92 and 0.83 with RMSEP values of 1.274 and 0.211 mg/g berry, respectively. The accuracy and precision of the prediction statistics were in line with the ones presented in the present work.

In terms of the computational time, the processing of the spectral measurements for each block (five consecutive vines) took approximately 5 minutes. This process, partly automated, divided in four steps (Section 4.4.1), involved two different software packages. The prediction of the unknown spectra using PLS models required less than 1 minute per block. Therefore, the total time needed to process the spectra and to predict these three grape composition parameters (TSS, total anthocyanins, and total polyphenols) per block would be 6 minutes, that is less than 1.5 minutes per vine. Compared to hyperspectral imaging, the contactless spectrometer provided the spectra directly, without the need of computationally expensive processing. This simpler nature of the acquired data accounts for a reduced computational time (around 3.6 hours for 36 blocks of five vines each vs 5.5 hours for 36 hyperspectral images [29]). Moreover, since both a light source and the reference (white and dark) measurements are enclosed in the VIS + SW − NIR system, in-field monitoring and data acquisition becomes less affected by environmental lighting conditions than measurements with a hyperspectral camera.

The potential of VIS + SW − NIR contactless spectroscopy has also been confirmed through the development of prediction maps for grape TSS, anthocyanins and total polyphenols concentrations and their evolution along the four different dates during the ripening process. The mapping of the grape composition parameters in the vineyard plot can be addressed to classify the vineyard plot into different grape composition zones during ripening and to determine the optimal timing of harvest in each delineated zone, enabling the winegrowers with a new monitoring tool towards improved and optimized decision-making. Moreover, the developed system provides spatial information on grape composition at each measuring date, which could not be achieved with the traditional manual sampling protocol of 100 to 200 berries.

One important point to take into account is the need to establish predictive relationships for entire cluster compositional characters based on the composition of visible berries as a first step towards the development of non-destructive methods, such as the one presented in this work to measure grape composition [36]. In this regard, the work of Tang et al. [36] concluded that variation in cluster compactness could contribute to variation in predictive relationships among varieties. Additionally, other factors, such as row orientation, or the level of defoliation at the fruiting zone, could potentially affect in a dissimilar way the grape metabolism of exposed vs non-exposed berries. Therefore, further research should be conducted involving vineyards planted with different varieties, row orientations, trellising, subjected to different climates to ensure the robustness of the predictive models using contactless VIS + SW − NIR spectroscopy on-the-go.

The remarkable outcomes obtained in this study reveal the actual applicability of bringing this non-destructive methodology based on spectroscopy from indoor applications to the field, either embedded in agricultural vehicles (during another viticultural operation, e.g., tilling) or mounted on phenotyping platforms or even robots to monitor agricultural crops directly in the field [37,38,39].

## 4. Materials and Methods

The experimental study involved the on-the-go acquisition of grape cluster spectra from the fruiting zone of the grapevine canopy using a spectral device for the estimation of chemical grape composition parameters at several dates during the grape ripening period. The regions of grape clusters scanned by the device were picked for the analysis of TSS, anthocyanins and total polyphenols concentrations. Grape cluster spectral measurements were filtered, averaged and analysed to be modelled using Partial Least Squares (PLS). The last step was the spatiotemporal evaluation of these composition parameters by mapping them during all the measurement dates.

### 4.1. Experimental Layout

The experiment was performed in a commercial vineyard located in Ábalos, La Rioja, Spain (Lat. 42° 34’ 45.7", Long. −2° 42’ 27.78", Alt. 628 m) during four dates from early August to late September 2017, along the grape ripening period. The vineyard was planted in 2010 with grapevines of (*Vitis vinifera* L.) Tempranillo, grafted on rootstock R-110. The vines were trained to a vertically shoot-positioned (VSP) trellis system on a double-cordon Royat with vine spacing of 2.20 m between rows and 1.0 m between vines in a northeast-southwest orientation.

With the purpose of ensuring an appropriate variability of grape composition, three different equally-distanced rows were selected and, within each one of them, 12 blocks with five plants each were chosen for the spectral and grape analyses, making up a total of 36 blocks, that were monitored during four dates from veraison to harvest. The four dates belong to different phenological stages according to the modified Eichhorn and Lorenz system [40]: 11 August, stage 36; 24 August, stage 37; 18 September, stage 38; and 28 September, stage 38.

The spectral measurements (a total of 144 throughout the whole experiment) were carried out on the east side of the canopy. This side was defoliated at the end of July, following a common viticultural practice in the region, to promote air circulation and sun exposure in the cooler, morning hours of the day. Hence, 36 blocks were measured each date, making a total of 144 measurements.

### 4.2. On-The-Go VIS+SW-NIR Measurements

On-the-go spectral measurements in the vineyard were acquired using a VIS + SW − NIR PSS 1050 spectrometer (Polytec GmbH, Waldbronn, Germany) operating in the 570–990 nm spectral range, at a 2 nm resolution, with 215 datapoints per spectrum.

The spectrometer was an active VIS + SW − NIR optical device with a polychromator as reflection light source selector, and Silicon (Si) detectors. The system includes a sensor head for light emission (by an integrated 20W tungsten lamp) and capturing, and a processing unit, both linked by an optical fiber.

**Figure 4:**
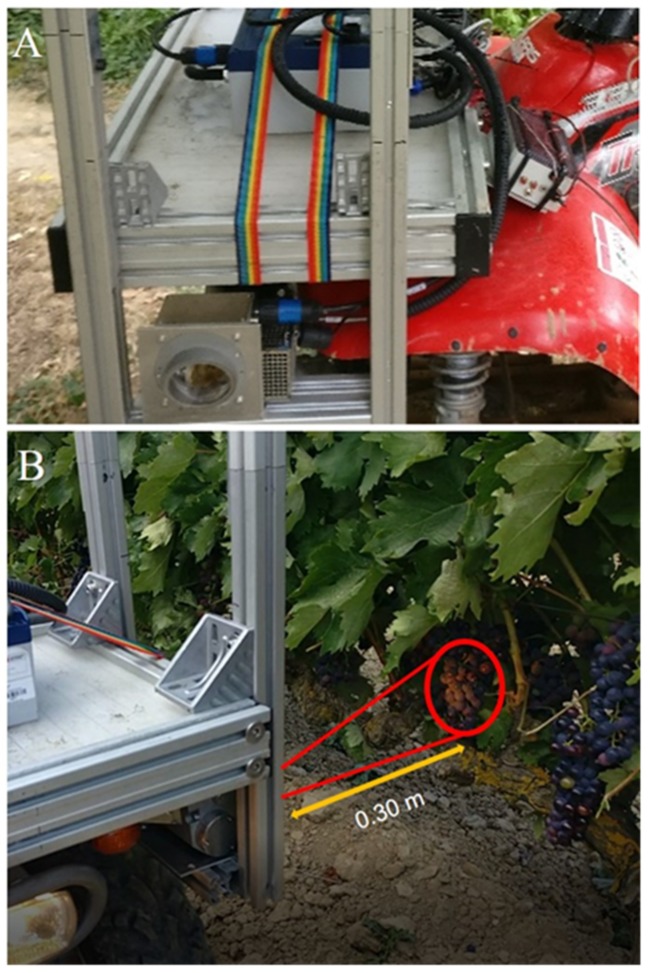
(**A**) Visible-Short Wave Near Infrared (VIS + SW − NIR) spectral acquisition system installed on the all-terrain vehicle (ATV) used for contactless on-the-go grape clusters spectral measurements in the vineyard. (**B**) Head sensor monitoring the grape cluster in motion.

The system was mounted in the front part of an all-terrain-vehicle (ATV) (Trail Boss 330, Polaris Industries, Minnesota, USA), aiming to the left and able to make spectral acquisitions controlled by a physical trigger while the ATV was in motion. The sensor head was placed at a height of 0.80 m from the ground, pointed to the canopy on a lateral point of view at 0.30 m of distance in order to cover the fruiting zone (Figure 4).

The circular measuring spot area had a diameter of around 1.9 cm (area of 2.83 cm^2^). On-the-go spectral measurements were acquired on the east canopy side at a constant speed of 5 km/h and rate of spectral acquisition of 18Hz. Spectral measurements were georeferenced using a GPS receiver Ag Leader 6500 (Ag Leader Technology, Inc., Ames, IA, USA) with RTK correction installed on the ATV.

### 4.3. Berry Composition Analysis

A total of 200 grape berries from all exposed clusters per block were collected and labeled immediately after the on-the-go VIS+SW-NIR measurements during the four dates.

The samples were transported to the laboratory of the University of La Rioja in portable refrigerators where they were stored in a freezer at −20 °C until chemical analysis. Once defrosted overnight in a cold room at 4 °C, a subsample of 100 berries was hand crushed and filtered. Total soluble solids (TSS), anthocyanins and total polyphenols concentrations were measured from the berries corresponding to each block. The TSS concentration was determined using a temperature compensating digital refractometer Quick-Brix 60 (Mettler Toledo, LLC, Columbus, OH, USA), expressed as °Brix. The remaining berry sub-sample was homogenized using a high-performance disperser T25 Ultra-Turrax (IKA, Staufen, Germany) at high speed (14,000 rpm for 60 s). Subsequently, anthocyanin and total polyphenols were analyzed following the Iland method [8]. Anthocyanin concentrations were expressed as mg/ fresh berry mass, whereas total polyphenols were expressed as absorbance units (AU) at 280 nm/ fresh berry mass.

### 4.4. Data Analysis

#### 4.4.1. Spectral Processing

**Figure 5:**
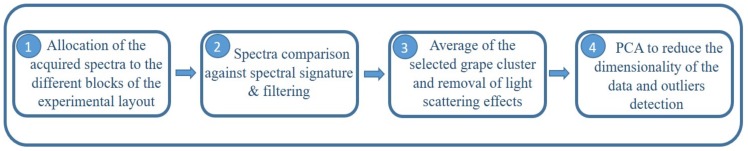
Design of the spectral processing procedure required to analyze on-the-go spectral measurements of grape clusters under field conditions.

The spectral processing followed four essential steps (Figure 5). The first one consisted on the allocation of the acquired spectra to the different blocks of vines within each equally-distanced rows in the field experiment. Within each block the raw on-the-go spectral measurements captured information from leaves, gaps, wood, metal, etc., so a filtering step to retain only the spectral information of grape berries was needed.

**Figure 6:**
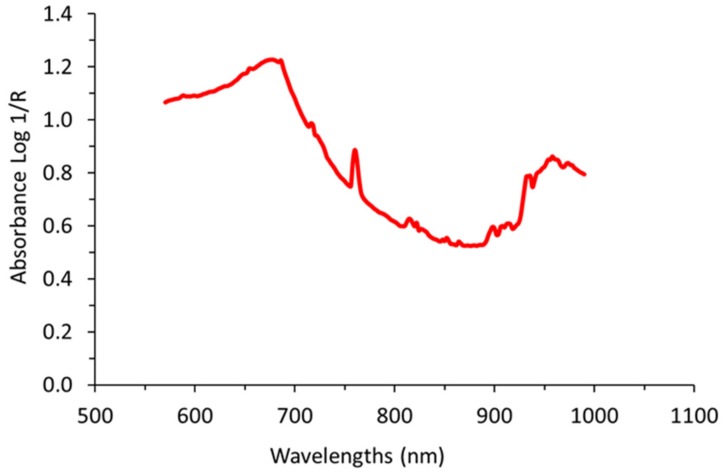
Spectral signature manually taken and averaged previous to on-the-go acquisitions from several grape clusters. This signature was used for filtering the on-the-go spectra and to select only those spectral signals belonging to grape clusters.

In order to retain those spectra corresponding to grape clusters, spectra comparison against manually-taken spectral signatures of grape clusters (Figure 6) was performed using the “Spectra Comparison & Filtering” tool from the SL Utilities software (version 3.1, Polytec GmbH, Waldbronn, Germany). Only spectra which passed the “Spectra Comparison & Filtering” thresholds were considered as valid to be used in calculating the average spectrum per block. The settings Cosine was the method used to adjust the threshold value to determine the required similarity of the raw on-the-go spectra to the defined signature of grape clusters spectrum. A higher threshold value (close to 1) means that greater similarity is required to accept the measured spectrum as a true grape berry spectrum. The threshold value in the field experiment was set to 0.993.

**Figure 7:**
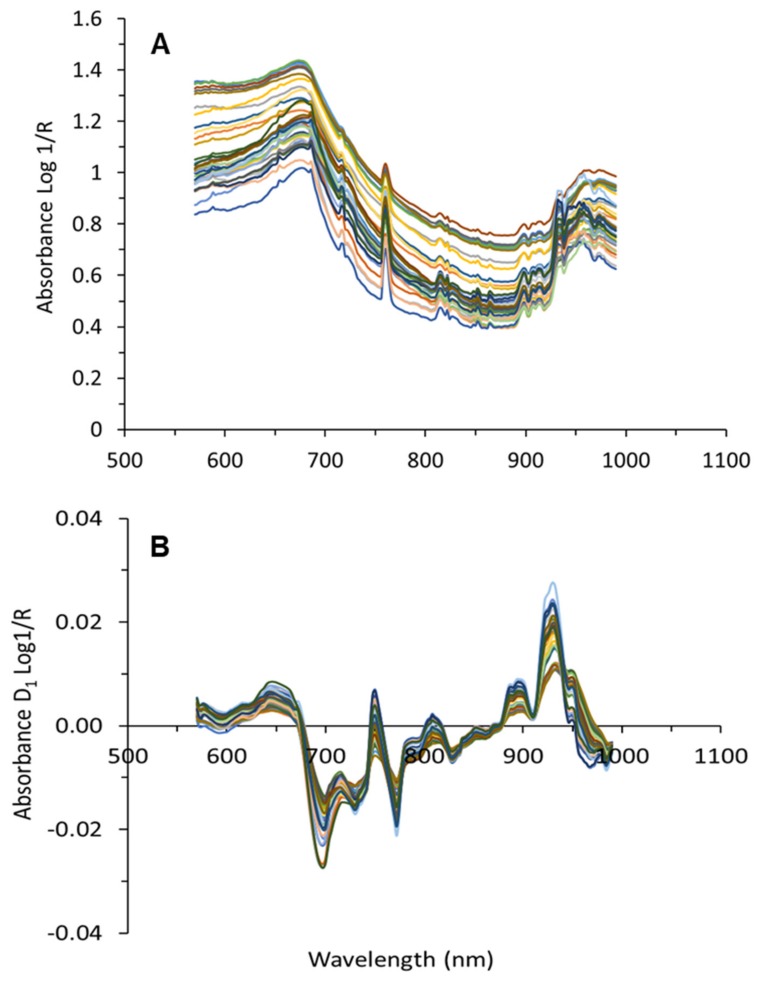
Average raw (**A**), and processed with Savitzky–Golay smoothing filtering (1st derivative, window size 15) (**B**) spectra collected on-the-go from grape clusters.

The third step involved the averaging of the filtered grape cluster spectra per block and removal of the effects of light scattering. Different combinations of several spectral pre-processing filters were tested. These filters involved the use of standard normal variate (SNV) [41,42] and the application of the Savitzky–Golay smoothing and derivative process [43], selecting different values for the window size and the degree of the derivative. Both SNV and Savitzky–Golay derivative techniques contributed to the removal of light scattering effects. Figure 7 shows the average absorbance raw (Figure 7A) and processed (after application of Savitzky–Golay first derivative) for grape cluster spectra from one date collected on-the-go.

In the last step principal component analysis (PCA) was used to reduce the dimensionality of the data structure, to visualize the presence of spectra outliers and also to identify the main sources of variability in the spectra [44,45]. Outlier detection was performed based on the Residuals (Q) and Hotelling values (T^2^) for the detection of samples with atypical spectra [46].

Spectral data manipulation and calibration models were performed with algorithms programmed in MATLAB (version 8.5.0, The Mathworks Inc., Natick, MA, USA). The partial least squares (PLS) Toolbox (version 8.1, Eigenvector Research, Inc., Manson, WA, USA) was used for principal component analysis (PCA) and partial least square regression (PLS).

#### 4.4.2. Calibration and Prediction Models

Once the grape cluster spectra were processed and the chemical grape compositional parameters were obtained for each block, they were used to build the dataset, in which each spectrum was linked with its corresponding berry composition analysis (TSS, anthocyanins and total polyphenols concentrations). Considering 12 blocks per three rows and four different measurement dates, the dataset comprised a total of 144 samples.

In order to train robust models, capable of predicting totally unknown samples, the original dataset, was split up into two independent randomized datasets: a calibration one (comprising 80% of all data), consisting of 116 samples, and an external validation (prediction) set, which comprised the remaining 28 samples (20%). Samples of both data sets were appropriately distributed and covered the entire range of the grape composition parameters. The calibration dataset was used to train and to perform an internal cross-validation of the model, while the external validation (prediction) set was only utilized for prediction purposes, using the calibration models.

Partial Least Squares (PLS) regression was used as the algorithm for training the grape composition parameters prediction models. This algorithm has proved to be an accurate, robust, and reliable chemometric method [47] to analyze spectral data, as it is capable to deal with a vast amount of data, especially when the number of attributes (wavelengths in this case) largely surpasses the number of samples. The input independent variables X were the 215 wavelengths within the spectral range of 570–1000 nm, while TSS, anthocyanins and total polyphenols concentrations were used as dependent variables Y, each one for the training of three different models.

The calibration dataset was used to train the model, and statistics of calibration and cross validation, using a 10-fold venetian blind approach, were computed to assess the performance of the built models. The optimal number of latent variables (LVs) was selected as the one yielding the lowest root mean square error of cross-validation (RMSECV). The validation dataset was never used in the training process. It was employed only for testing with external samples, also called external validation or prediction. To evaluate the quality of the obtained models, the determination coefficient of calibration (R^2^_c_), cross-validation (R^2^_cv_), and prediction (R^2^_p_), the root mean square error of calibration (RMSEC), cross-validation (RMSECV) and prediction (RMSEP) were calculated.

### 4.5. Mapping

Based on the developed prediction models, prediction maps of TSS, anthocyanins and total phenol concentrations were created to monitor and illustrate the spatial variability of a commercial vineyard’s grape composition using VIS + SW − NIR spectroscopy during the ripening period. Multilevel b-spline interpolation [48] with QGIS 2.18 (Free Software Foundation, Boston, MA, USA) was used to carry out the mapping tasks.

## 5. Conclusions

VIS + SW − NIR technology has proven to be a real alternative to appraise and map the vineyard grape composition variability in VSP vineyards, with a high spatial and temporal resolution, in a fast and non-destructive way. The capability to monitor the spatiotemporal evolution and distribution of total soluble solids, anthocyanins, and total polyphenols along the grape ripening process in a commercial vineyard will greatly enhance the decision-making about differential fruit allocation and harvest according to grape composition and quality.

## Figures and Tables

**Table 1:** Calibration, cross-validation, and external validation (prediction) of the best models obtained to predict the total soluble solids, anthocyanins and total polyphenols concentrations in grape clusters under field conditions from on-the-go Vis + SW − NIR spectroscopy (570–990 nm).

						Calibration		Cross-Validation		External Validation	
Parameters	Spectral Treatment	N	SD	Range	PLS Factor	RMSEC	R^2^_c_	RMSECV	R^2^_cv_	RMSEP	R^2^_p_
**Total soluble solids** (°**Brix**)	D1W15	116	4.403	10.70–25.20	7	1.119	0.93	1.248	0.92	1.011	0.95
**Anthocyanins** **(mg/berry)**	D1W15	116	1.329	0.09–4.64	6	0.607	0.79	0.664	0.75	0.618	0.79
**Total polyphenols** **(Au/berry)**	SNV + DT D1W15	116	0.947	0.14–4.70	7	0.642	0.54	0.728	0.42	0.749	0.43

SNV: standard normal variate. DnWm, Savitzky–Golay filter with n-degree derivative, window size of m. N: number of samples used for calibration and cross-validation models after outlier detection. SD: standard deviation. RMSEC: root mean square error of calibration. R^2^_c_: determination coefficient of calibration. RMSECV: root mean square error of cross-validation. R^2^_cv_: determination coefficient of cross-validation. RMSEP: root mean square error of prediction. R^2^_p_: determination coefficient of prediction.
